# Supplementary material for: Fluorescent, Prussian Blue-Based Biocompatible Nanoparticle System for Multimodal Imaging Contrast
Source: Nanomaterials (Basel). 2020 Aug 31;10(9):1732. doi: 10.3390/nano10091732 (PMC7557721; doi:10.3390/nano10091732)
Supplement: Supplementary file 1 [file nanomaterials-10-01732-s001.pdf]

nanomaterials

Supplementary Materials

Fluorescent, Prussian Blue-Based Biocompatible Nanoparticle System for Multimodal Imaging Contrast

László Forgách [1,*], Nikolett Hegedűs [1], Ildikó Horváth [1], Bálint Kiss [1], Noémi Kovács [1], Zoltán Varga [1,2], Géza Jakab [3], Tibor Kovács [4], Parasuraman Padmanabhan [5], Krisztián Szigeti [1,*,†] and Domokos Máthé [1,6,7,*,†]

1 Department of Biophysics and Radiation Biology, Semmelweis University, 1085 Budapest, Hungary; hegedus.nikolett@med.semmelweis-univ.hu (N.H.); horvath.ildiko@med.semmelweis-univ.hu (I.H.); kiss.balint@med.semmelweis-univ.hu (B.K.); kovacsnoi@hotmail.com (N.K.); varga.zoltan@ttk.mta.hu (Z.V.)
2 Institute of Materials and Environmental Chemistry, Research Centre for Natural Sciences, 1117 Budapest, Hungary; varga.zoltan@ttk.mta.hu (Z.V.)
3 Department of Pharmaceutics, Semmelweis University, 1085 Budapest, Hungary; jakab.geza@pharma.semmelweis-univ.hu
4 University of Pannonia, Institute of Radiochemistry and Radioecology, 8200 Veszprém, Hungary; kt@almos.vein.hu
5 Lee Kong Chian School of Medicine, Nanyang Technological University, 636921 Singapore, Singapore; ppadmanabhan@ntu.edu.sg
6 In Vivo Imaging Advanced Core Facility, Hungarian Centre of Excellence for Molecular Medicine, 6723 Szeged, Hungary
7 CROmed Translational Research Centers, 1047 Budapest, Hungary
* Correspondence: forgach.laszlo@med.semmelweis-univ.hu (L.F.); szigeti.krisztian@med.semmelweis-univ.hu (K.S.); mathe.domokos@med.semmelweis-univ.hu (D.M.); Tel.: +36-1-459-1500/60164 (L.F.); +36-1-459-1500/60210 (D.M.)
† These authors contributed equally to this work.

Native, citrate-coated PBNPs

The mean hydrodynamic diameter (intensity-based harmonic means or z-average) of citrate-coated PBNPs was 29.30 ± 2.08 (average ± SD, $n = 8$), as determined by DLS. This had only changed slightly with time. There was no significant colloidal alteration during the 8-week study; as the calculated 0.208 ± 0.015 polydispersity index (PDI) shows the PBNPs did not flocculate or aggregate during this time [9], (data not illustrated).

Preliminary fluorescent labelling studies

Our preliminary result suggested that for the fluorescent labelling of PBNPs, MB would be a suitable dye. We also experimented using other xanthene dyes e.g. Fluoresceine (S.1. (a)), Rhodamine B (S.1.(c)) and Eosine Y (S1.(b)).

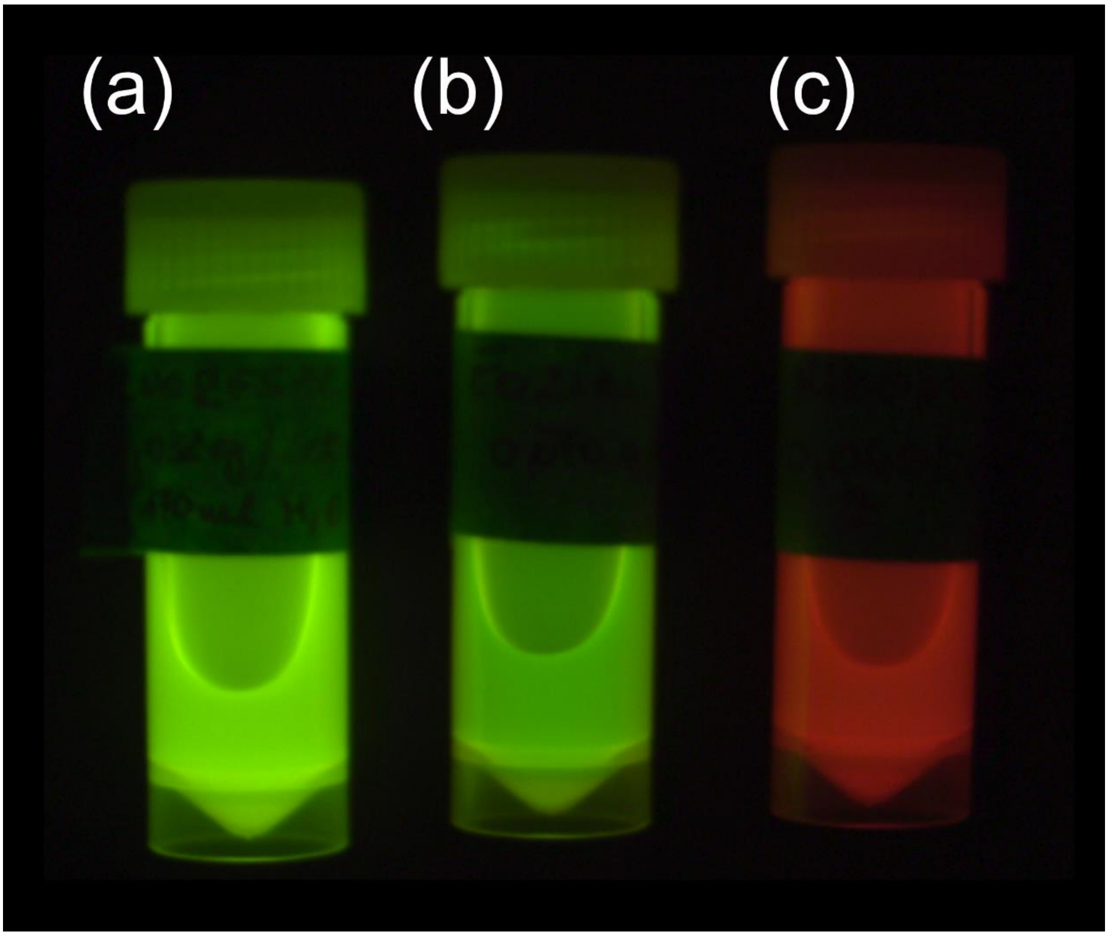

Figure S1 (**a**) Fluoresceine solution, imaged in the FOBI device. (**b**) Eosine Y solution, imaged in the FOBI device. (**c**) Rhodamine B image. FOBI: Fluorescent Organism Bioimaging Instrument

Thus, not all samples were amenable to fluorescent imaging in vivo, furthermore, they lacked stability during the early inspections. The aggregation of the nanosystem along with fluorescent dye wash-off was noted. The results are summarized in Table S2.

Table S1**.** Previous approaches made for labelling PBNPs with fluorescent dyes. "+" means successful step in the labelling, "-" stands for unsuccessful steps. "+ / -" successful step but negative result during the imaging measurement.

	**Fluoresceine**	**Rhodamine B**	**Eosine Y**	**Methylene blue**
Solubility	+ / -	+	+	+
Labelling	-	+ / -	+	+
In vitro fluorescence	-	-	+	+
Stability	-	-	+	+
In vivo measurement	-	-	+ / -	+

Fluorescein labelling of PBNPs was not successful. Experimenting with other dyes we found that Rhodamine B, Eosine Y and Methylene blue sorption to PBPNs was feasible. However, Rhodamine B labelled PBNPs lacked stability: immediately after labelling, aggregation was visible. The long-term stability of Eosine Y and MB labelled PBNPs and their in vivo imaging signalling performance was thus further investigated (Table S2.)

In vivo, SNR of Eosine Y labelled nanoparticles proved to be insufficient for evaluable result; however, Figure S2 shows the ex vivo distribution of the particles. Hyperintense changes, mainly due to the PBNP treatment are visible in the liver and gastro-intestinal tract; the autofluorescence of the skin is also seen at this dye`s main emission wavelength, further impeding the use of Eosine Y.

Non-pegylated PBNPs had a good imageable contrast but eliminating them too fast did not prove to be advantageous for in vivo applications.

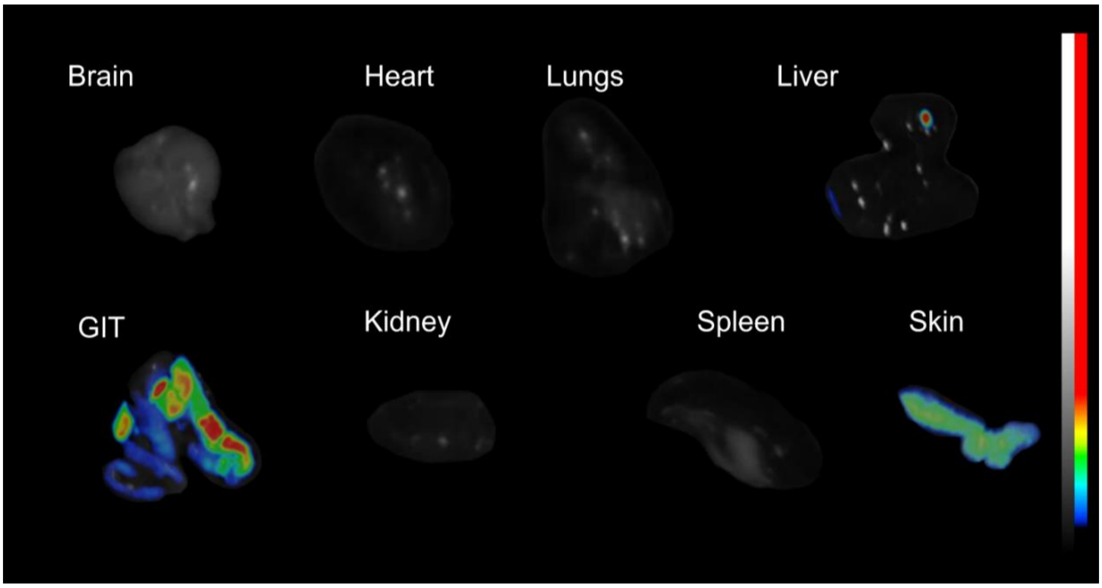

Figure S2. Ex vivo results of Eosine Y labelled PBNPs. (FOBI image, excitation 460-520 nm; cut off filter was permeable to the emission spectrum of the dye; exposure time: 1,000 msec; gain: 1). PBNPs: Prussian Blue nanoparticles; FOBI: Fluorescent Organism Bioimaging Instrument.

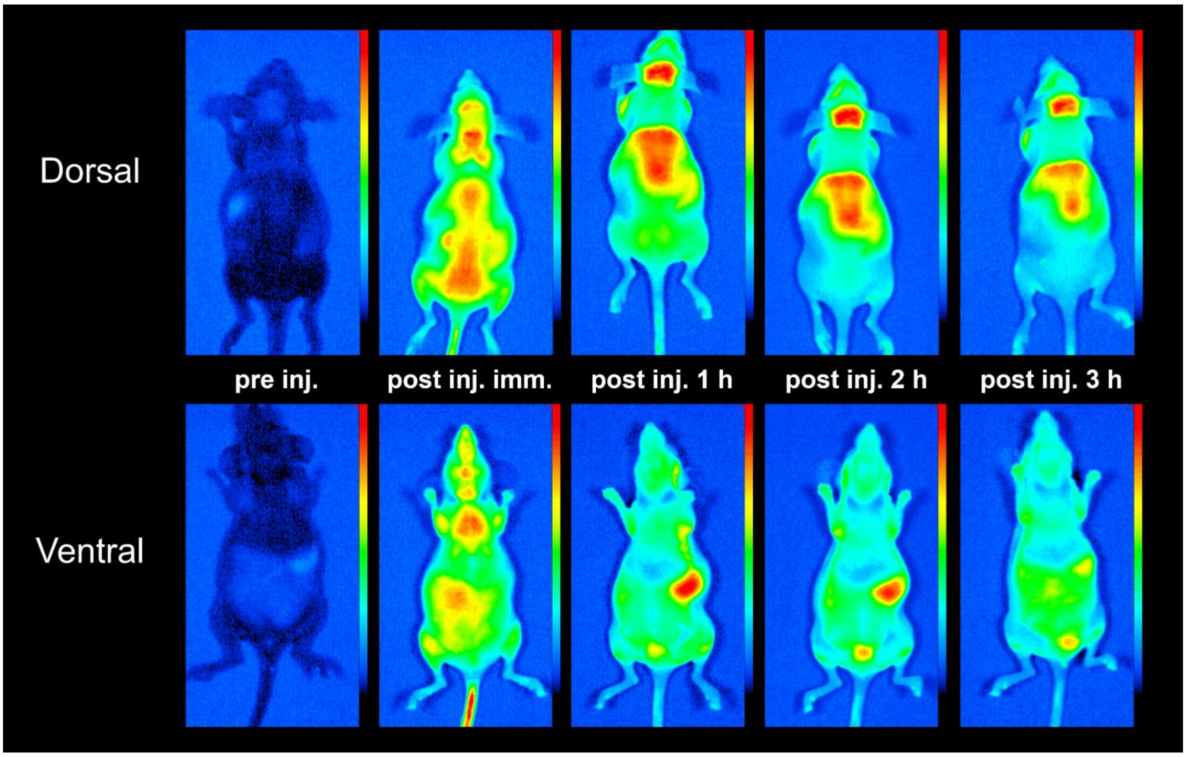

Figure S3. Methylene blue labelled unpegylated PBNPs, in vivo. (FOBI image; excitation 630-680 nm; cut off filter was permeable to the emission spectrum of the dye; exposure time: 1,000 msec; gain: 1). PBNPs: Prussian Blue nanoparticles; FOBI: Fluorescent Organism Bioimaging Instrument.

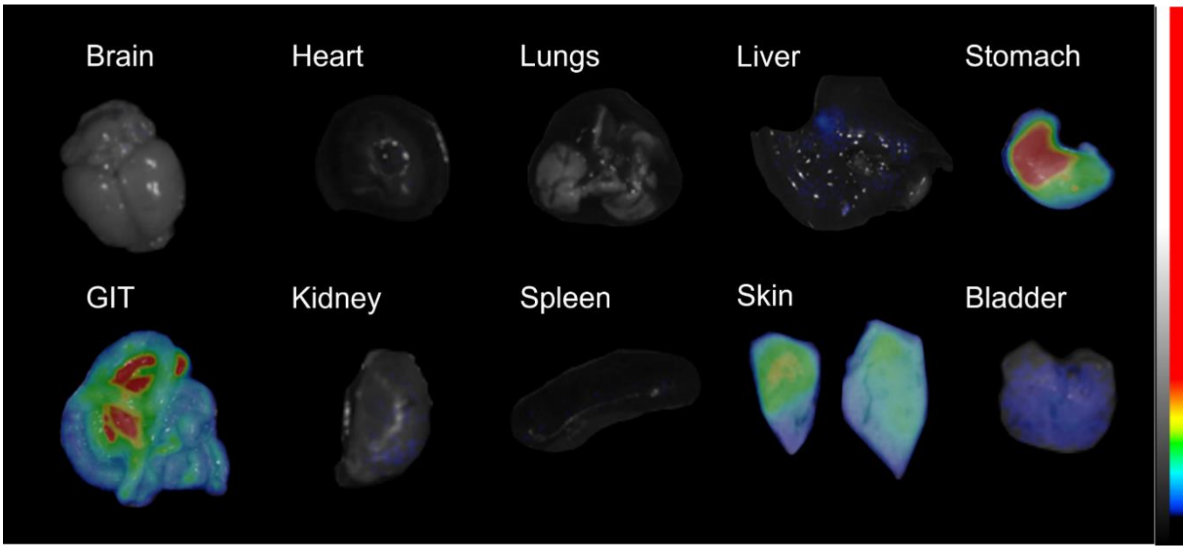

Figure S4. Methylene blue labelled PBNPs, ex vivo (FOBI image; excitation 630-680 nm; exposure time: 1,000 msec; gain: 1). Nanoparticles are present in the liver, stomach, gastro-intestinal tract and skin. PBNPs: Prussian Blue nanoparticles; FOBI: Fluorescent Organism Bioimaging Instrument.

Table S2. The detailed data of the preformulation measurements. The PEG content is displayed in mg and w/w%; the mean particle diameter by particle number is displayed in column four. The further investigations were not only based on the DLS data, but also on the macroscopic investigations, which, despite the promising results in the particle diameter, showed a controvesal image in several cases. However, the particle diameter was small, the whole sample showed a slight aggregation, which prevented further investigations and modifications of the particles (by fluorescent labelling).

	**PEG content / mg**	**PBNP to PEG / w/w%**	**Mean particle diameter / nm**
PEG 3000	0.75	133.33	24.06 ± 2.33
	1	100	25.13 ± 1.06
	1.25	80	23.35 ± 1.21
	2	50	28.54 ± 0.86
	5	20	28.06 ± 3.05
PEG 6000	7.5	13.33	34.84 ± 0.51
	10	10	24.43 ± 0.57
	12.5	8	22.71 ± 1.43
	20	5	24.42 ± 3.22
PEG 8000	10	10	24.58 ± 2.56
	12.5	8	22.20 ± 1.80

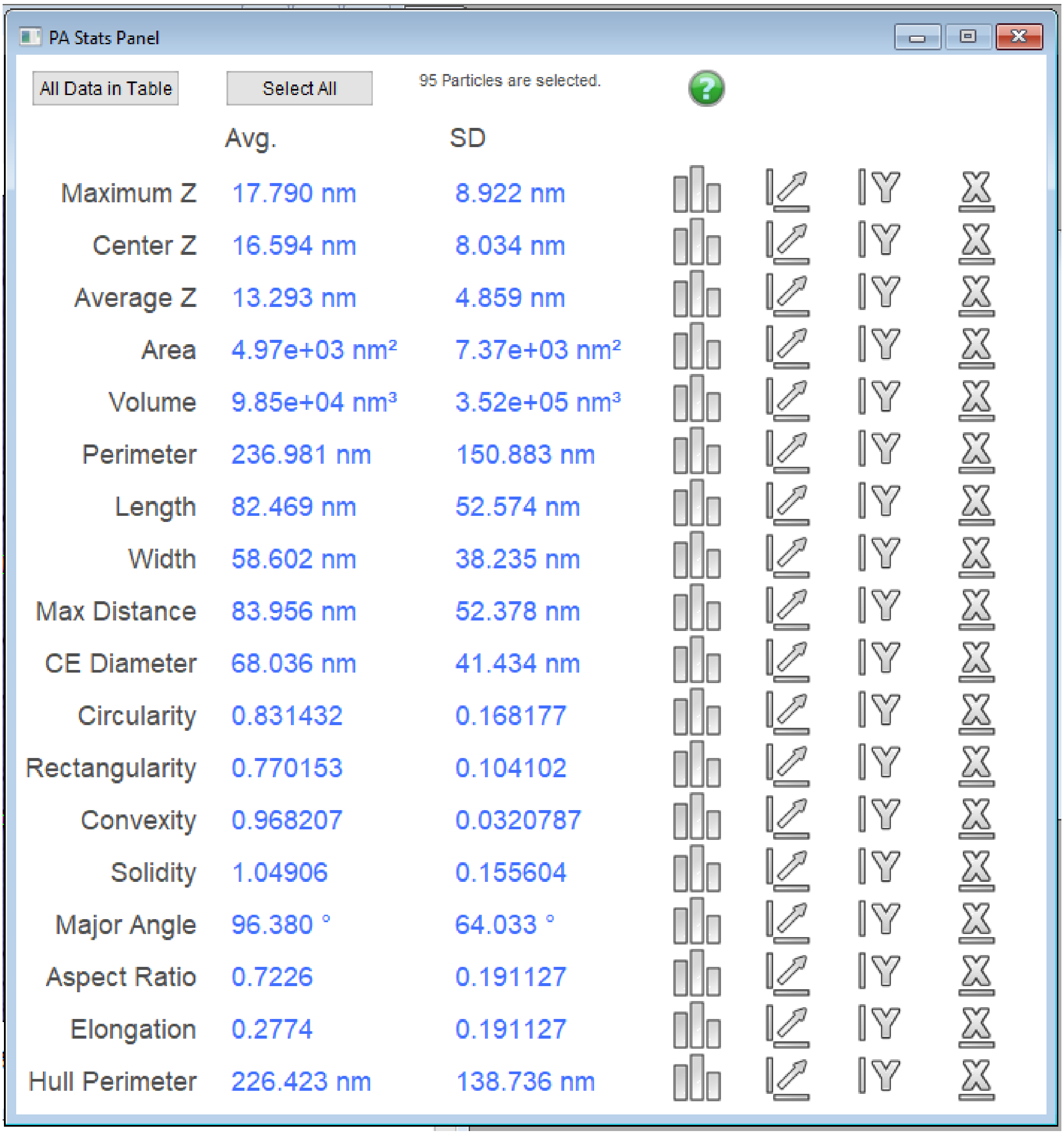

PA Stats Panel

All Data in Table | Select All | 95 Particles are selected.

	Avg.	SD
Maximum Z	17.790 nm	8.922 nm
Center Z	16.594 nm	8.034 nm
Average Z	13.293 nm	4.859 nm
Area	4.97e+03 nm²	7.37e+03 nm²
Volume	9.85e+04 nm³	3.52e+05 nm³
Perimeter	236.981 nm	150.883 nm
Length	82.469 nm	52.574 nm
Width	58.602 nm	38.235 nm
Max Distance	83.956 nm	52.378 nm
CE Diameter	68.036 nm	41.434 nm
Circularity	0.831432	0.168177
Rectangularity	0.770153	0.104102
Convexity	0.968207	0.0320787
Solidity	1.04906	0.155604
Major Angle	96.380 °	64.033 °
Aspect Ratio	0.7226	0.191127
Elongation	0.2774	0.191127
Hull Perimeter	226.423 nm	138.736 nm

Figure S5. The detailed data of AFM measurement.

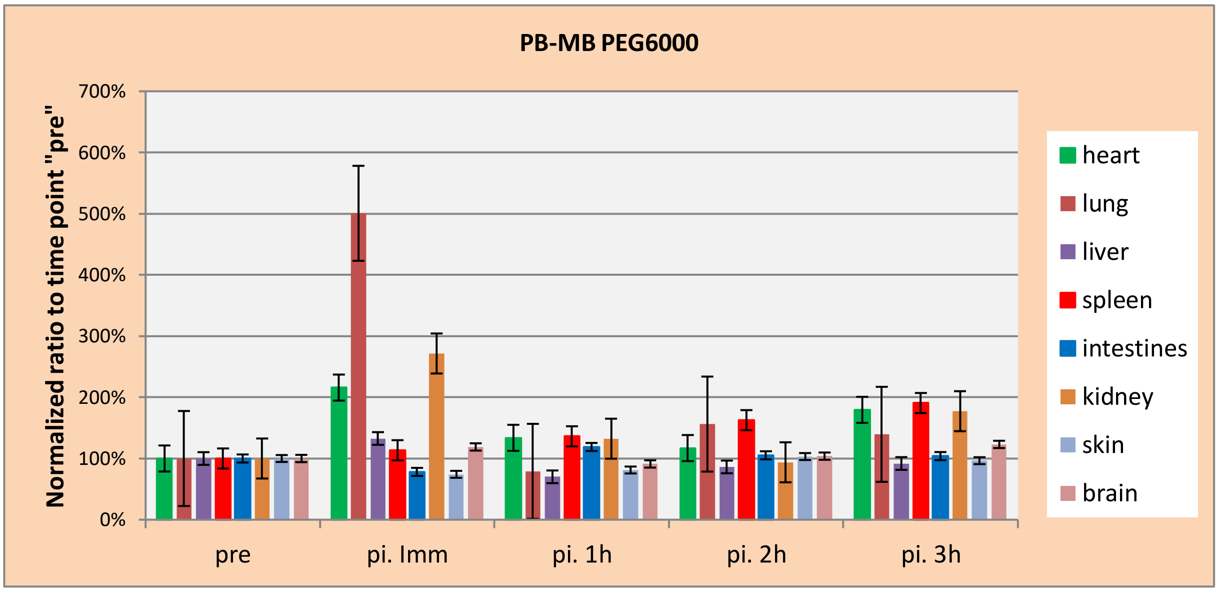

Figure S6. The kinetics of MB labelled pegylated PBNPs pre injection; post injection immediately; 1, 2, and 3 hours post injection.

Percent values (± SD) were based on the normalized fluorescent intensities pre injection. Significant uptake can be observed in the lungs (500 % ± 85 %; then 150 % ± 60 %) and spleen (162 % ± 18%), while a slight increase in fluorescent intensity in the intestines (107 % ± 5 %), along with the urinary excretion (270% ± 32 %; then 182 % ± 20 %). These results suggest that the MB and PBNPs were connected, their clearance followed the both characteristic pathways for MB and PBNP; furthermore, the PEG shell alone did not promote the renal excretion significantly. The fluorescent intensity in the heart, liver, and spleen suggest a prolonged circulation time.

X-Ray diffraction (XRD)

1000 μL of the undiluted suspensions of the samples were dried to a zero-diffraction plate, using infrared light. X-ray diffractograms were recorded by Philips PW 1810/1870 diffractometer applying monochromatized CuKα radiation (40 kV, 35 mA), scan speed of 1 s/step and a step size of 0.04°, between the range of 2θ = 10° - 50°. The evaluation of the diffractograms was carried out using Origin 9.0 (OriginLab).

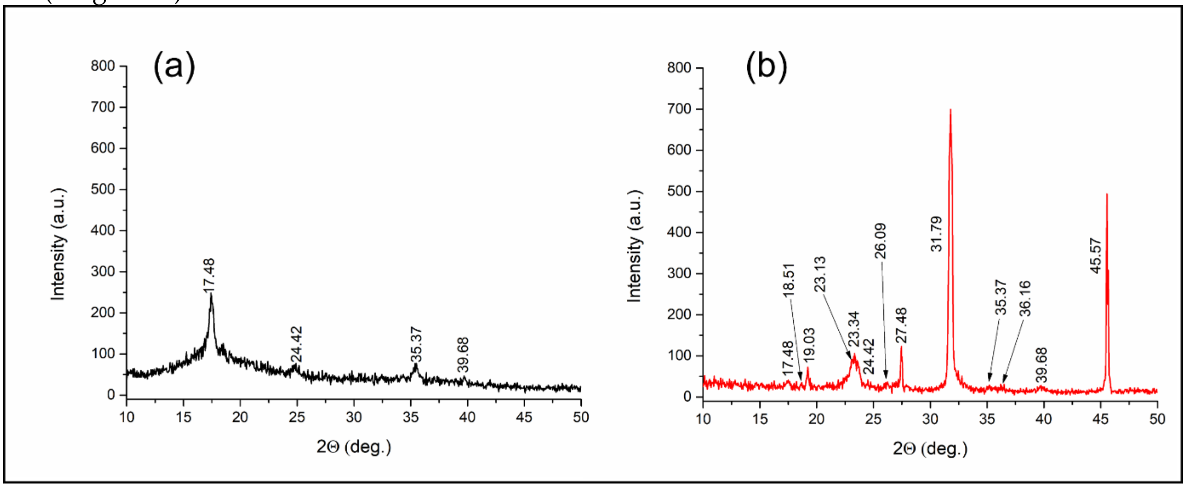

Figure 7. (**a**) The XRD-diagram of the unmodified PBNPs (2θ = 10-50°). (**b**) The XRD diagram of the modified PBNPs. XRD: X-ray diffraction; PBNPs: Prussian Blue nanoparticles.

Results - X-Ray diffraction (XRD)

X-ray diffraction is based on the elastic scattering of x-rays from structures that have a long-range order. It discovers the geometry or shape of a molecule. The two samples ((a)Prussian blue reference solution, which was used later for pegylation and methylene blue labelling and (b) pegylated, fluorescent labelled PBNPs) were measured. The spectra (Fig. S7 (a)) show diffraction peaks at 2θ = 17.480, 24.420, 35.370 and 39.680 which can be allocated to the Prussian blue phase crystal planes respectively. These peaks can be related to face-centered cubic structure of PB with space group Fm3m. Figure 3 (b) shows the existing peaks characteristic for PBNPs, furthermore, peaks related to PEG 6000 (2θ = 18.510, 19.030, 23.130, 23.340 and 26.090) appeared, along with the characteristic peaks of Methylene blue (2θ = 27.480, 31.790, 36.160 and 45.570) [59-62].

Fourier Transformation Infrared Spectroscopy (FT-IR)

The infrared spectra were recorded with a Bruker Vertex80v (Bruker Optics, Billerica, MA) FTIR spectrometer equipped with a high sensitivity MCT (mercury-cadmium-telluride) detector. Each spectrum was collected averaging 128 scans at 2 cm− 1 resolution. The infrared beam was focused on a high-pressure diamond anvil cell (Diacell, Leichester, UK) using a Bruker A525 type beam condenser. Three main measurement methods were used:

- 50 μL of the undiluted suspensions of the samples were dried to the detector, using compressed air. The measurements were executed under N2 atmosphere, to eliminate external humidity and water condensation at the detector (Figure S8/I).
- The samples were lyophilized, then the completely dry samples were measured in the diamond anvil cell (Figure S8/II).
- Freeze-dried samples were resolved in D2O (20 μL) and then dried to the detector, using compressed air. The measurements were executed under N2 atmosphere, to eliminate external humidity and water condensation at the detector (Figure S8/II).

Results - Fourier Transformation Infrared Spectroscopy (FT-IR)

The FTIR absorption spectra (Figure S8/I) give a comparison between unmodified PBNPs (Figure S8/I (a)) and the PEG 6000 and Methylene blue modified PBNPs (Figure S8/I (d)). In addition, the spectra of PEG 6000 (c) and MB (b) are shown, ranging from 400 cm-1 to 4,000 cm-1.

On the spectra of the pure and modified PBNPs, the peaks at 2,090 cm-1 and 494 cm-1 correspond to the stretching vibration of C≡N and Fe-CN-Fe, which are the typical signals of PB. Typically, peaks over 3,200 cm-1 are ascribed to O-H group, indicating the presence of interstitial water in the PB. (Figure S8/I (a-d)).

Figure S8/I (d) shows that the bands shift to lower wavenumbers. Furthermore, the peaks of PEG 6000 (c) and MB (d) could be identified, indeed, after the hypsocromic shift.

Figure S8/I (c) shows the absorption spectrum of PEG 6000. A broad band at 3,300 cm-1 is present, which can be related to an O-H stretching mode while the band at 1,650 cm-1 is a H-O-H bending vibration. A characteristic peak can be determined at 1,100 cm-1, which shows the C-O bonds.

Figure S8 (b) represents the absorption spectra of Methylene blue. Characteristic peaks can be observed at 3,265, 2,114, 1,633 and 585 cm-1.

On Figure S8/II (a), characteristic peak of PBNPs are present at 2,090 cm-1, which corresponds the stretching vibration of C≡N. The rehydrated sample binds also D2O, namely a peak is present above 2,7000 cm-1, which is related O-H (or O-D) stretching mode while the wider band (compared to the completely dry sample) at 1,650 cm-1 is a H-O-H (or D-O-D) bending vibration. Aromatic groups and methly groups are visible on Figure S8/II (b). Aromatic groups and methly groups are visible at 2807 and 2710 cm-1 represent the stretching vibration of -CH- and -CH3, aromatic vibrations are present from 1601-1372 cm-1, while the vibrations of the C=C skeleton of the dye are present at 1171 cm-1 (SI Figure S8 (b)).

The spectra of PEG 6000 (Figure S8/II (c)) shows wide bands at ~3500 cm-1; these are related to the -OH, and the -C-O stretching is clearly visible ~1200 cm-1.

On the other hand, Figure S8 (d) has a visible vibration band around 1170 cm-1, which could represent the C=C skeleton of the aromatic rings. Also, signs of C≡N bands are present at 2090 cm-1, and the board band of -OH around 3500 cm-1 and -C-O stretching around 1200 cm-1. These vibration and stretching bands can be related to both citric acid and PEG, as well was PB itself.

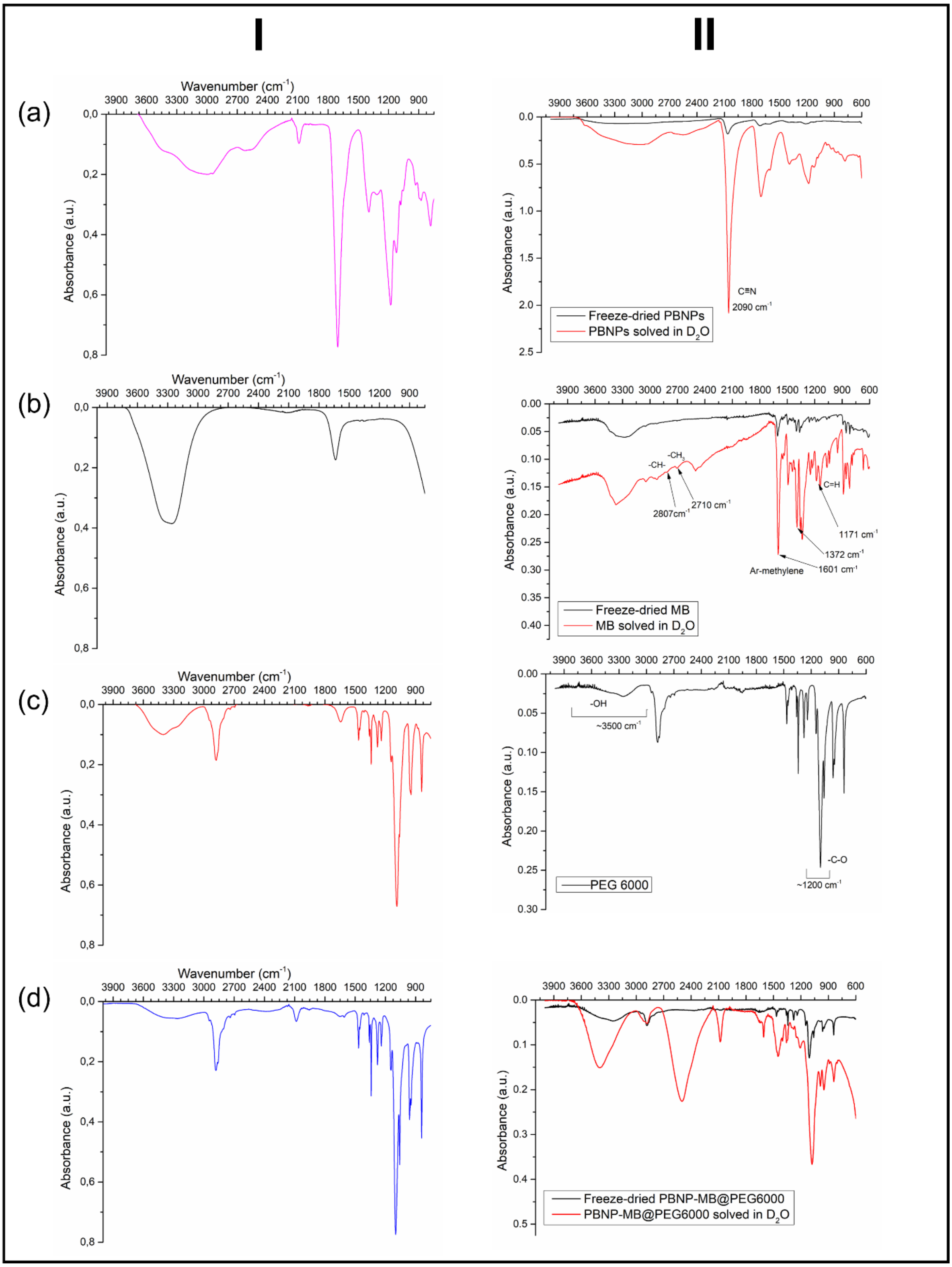

Figure 8. I (**a**) FT-IR spectra of unmodified PBNP (mid-IR region = 4,000-750 cm-1). (**b**) FT-IR spectra of Methylene blue (mid-IR region = 4,000-750 cm-1). (**c**) FT-IR spectra of PEG 6000 (mid-IR region = 4,000-750 cm-1). (**d**) FT-IR spectra of pegylated, fluorescent PBNPs (mid-IR region = 4,000-750 cm-1). **Figure 8. II** (a) the FT-IR spectra of unmodified PBNPs. (b) FT-IR spectra of Methylene blue. (**c**) FT-IR spectra of PEG 6000. (**d**) FT-IR spectra of pegylated, fluorescent PBNPs. FTIR: Fourier-transform infrared spectroscopy; PBNPs: Prussian Blue nanoparticles; PEG 6000: polyethylene glycol.

References in Supplemental Information:

59. PDF, I. (2018). 4+ 2019. Kabekkodu, S.; Ed. International Centre for Diffraction Data (ICDD): Newtown Square, PA 19073, USA
60. Milošević, M.D.; Logar, M.M.; Poharc-Logar, A.V.; Jakšić, N.L. Orientation and optical polarized spectra (380–900 nm) of methylene blue crystals on a glass surface. Int. J. Spectrosc. 2013, doi:10.1155/2013/923739.
61. Canossa, S.; Bacchi, A.; Graiff, C.; Pelagatti, P.; Predieri, G. Structural investigation by X-ray diffraction analysis on the behaviour of methylene blue towards transition metal anions. International School of Crystallography, 48th Course, 2015. Available online: https://www.researchgate.net/publication/281862069, (accessed 5 June 2015).
62. Prasanthi, D.; Jagadish, G.; Aishwarya, K.L. Solid dispersions of fenofibrate: Comparision of natural and synthetic carriers. The Pharma Innovation Journal 2019. Available online: http://www.thepharmajournal.com/archives/2019/vol8issue3/PartA/8-1-133-661.pdf (accessed 25 February 2019).
